# G908R NOD2 variant in a family with sarcoidosis

**DOI:** 10.1186/s12931-018-0748-5

**Published:** 2018-03-20

**Authors:** Valérie Besnard, Alain Calender, Diane Bouvry, Yves Pacheco, Catherine Chapelon-Abric, Florence Jeny, Hilario Nunes, Carole Planès, Dominique Valeyre

**Affiliations:** 1 Université Paris 13, Sorbonne Paris Cité, Laboratoire EA2363 “Hypoxie et Poumon”, 74 rue Marcel Cachin, 93017 Bobigny cedex, France; 2 Génétique des cancers et maladies multifactorielles, Hospices Civils de Lyon, GHE, Centre de Biologie et Pathologie ES, Bron, France; 3 AP-HP, Hôpital Avicenne, Bobigny, France; 4 Hospices Civils de Lyon, Centre Hospitalier Lyon Sud, Université Claude Bernard - Lyon 1, EA-7426 Lyon, France; 5 Université Claude Bernard Lyon 1 - EA-7426, 165 Chemin du Grand Revoyet, F-69495 Pierre Benite, France; 6 Department of Internal Medicine and Clinical Immunology, Groupe Hospitalier La Pitié-Salpêtrière, Assistance Publique-Hôpitaux de Paris (AP-HP), Paris, France

**Keywords:** Familial sarcoidosis, NOD2, Genetic susceptibility

## Abstract

**Background:**

Sarcoidosis is a systemic disease characterized by the formation of immune granulomas in various organs, mainly the lungs and the lymphatic system. Exaggerated granulomatous reaction might be triggered in response to unidentified antigens in individuals with genetic susceptibility. The present study aimed to determine the genetic variants implicated in a familial case of sarcoidosis.

**Methods:**

Sarcoidosis presentation and history, NOD2 profile, NF-κB and cytokine production in blood monocytes/macrophages were evaluated in individuals from a family with late appearance of sarcoidosis.

**Results:**

In the present study, we report a case of familial sarcoidosis with typical thoracic sarcoidosis and carrying the *NOD2 2722G > C* variant. This variant is associated with the presence of three additional SNPs for the *IL17RA*, *KALRN* and *EPHA2* genes, which discriminate patients expressing the disease from others. Despite a decrease in NF-κB activity, *IL-8* and *TNF-A* mRNA levels were increased at baseline and in stimulated conditions.

**Conclusions:**

Combination of polymorphisms in the *NOD2*, *IL17RA*, *EPHA2* and *KALRN* genes could play a significant role in the development of sarcoidosis by maintaining a chronic pro-inflammatory status in macrophages.

**Electronic supplementary material:**

The online version of this article (10.1186/s12931-018-0748-5) contains supplementary material, which is available to authorized users.

## Background

Sarcoidosis is a systemic granulomatous disease of unknown cause. A genetic predisposition is suggested by familial histories and the risk for the disease multiplied by 80 in monozygotic twins of index cases [1, 2]. Genome-wide association studies and candidate gene-driven investigations identified several genetic risk foci for sarcoidosis, including butyrophilin-like 2 gene [3–5], Annexin 11 [6], a locus on chromosome 11q13.1 [7], several loci in the HLA region [8] and in the IL23/Th17 signalling [9]. *NOD2* mutations have been evidenced in granulomatous diseases, including Blau syndrome (BS), early onset sarcoidosis (EOS) and Crohn’s disease (CD). NOD2 is a cytosolic protein involved in sensing microbial cell wall components and regulating inflammatory processes by activating NF-κB [10]. A deficit in sensing bacteria in monocytes/macrophages might result in an exaggerated inflammatory response by the adaptive immune system. Thus *NOD2* mutations are considered to play a major role in the pathogenesis of granulomatous diseases [11, 12]. Variants may concern different NOD2 domains, NACHT domain for BS and EOS and LRR domain for CD [11, 13]. NACHT domain variants enhanced NOD2 activity, whereas LRR domain variants reduced NOD2.

Although scientific evidence was provided indicating that the NOD2 signalling pathway was involved in sarcoidosis pathogenesis, selection of patients with confirmed sarcoidosis from the Sarcoidosis Genetic Analysis study population [14] and the ACCESS study [15] with or without joint and skin involvement showed no evidence of *NOD2* mutation [16]. Up to now, no study has evidenced any *NOD2* mutation in sarcoidosis with a late typical presentation. The role of the *NOD2* gene in sarcoidosis was associated with increase susceptibility for developing sarcoidosis [17, 18].

The SARCFAM project is a French national project on familial sarcoidosis which allowed the recruitment of more than 180 families with at least two first-degree affected members [19]. Screening of nine sarcoidosis by WES has provided extensive and complex data which are included in a larger program of the SARCFAM project. Among this series, a subset of 10 families gathered more than 3 patients. One of them referred as “X” was of particular interest due to the identification of NOD2 variants, including the G908R mutation that has been described in Crohn’s disease. Identification of the *NOD2* variant in the LRR domain (G908R in exon 8) was confirmed by Sanger sequencing.

In the present study, we report sarcoidosis presentation and history, NOD2 profile and NF-κB and cytokine production in blood monocytes in affected patients of the family X and to compare genetic and NF-κB and cytokine profiles with members of the family unaffected by sarcoidosis and safe controls.

## Methods

### Populations

**Fig. 1:**
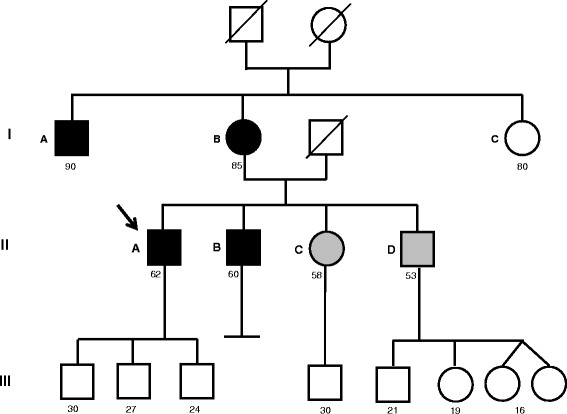
Pedigree of Family X with cases of pulmonary sarcoidosis. Circles represent females; squares represent males. Slashed symbol indicate deceased subjects. Individuals with confirmed sarcoidosis are indicated in black symbols. Relatives in grey symbols underwent a workup diagnosis (clinical examination, blood tests and chest X ray) that allowed to exclude sarcoidosis. The current age is indicated below each symbol. Numbers beside each symbol correspond to the individuals described in Table 1. The directional arrow indicates the index case. His maternal grand-mother died in 1958. She was known to have recurrent asthma attacks. No medical file is available for this woman

The study received institutional review board approval (CPP IRN number 00009118) according to French legislation. Written informed consent for all participants was obtained for genetic and biological investigations. Three groups were studied:

*Members of family X with confirmed sarcoidosis (n = 4)* (Table 1 and Fig. 1). Sarcoidosis diagnosis was made according to sarcoidosis statement [20]. Three of them could be investigated (IB, IIA and IIB). In them, clinical presentation was typical, evidence of non caseating granulomas was obtained and there was no element in favour of any alternative granulomatous disease. None of them presented any element in favour of BS, EOS nor CD. In particular, there was no digestive tract history except for one patient with colic polyps who underwent twice colonoscopy without evidence of macroscopic or histologic arguments for CD.

**Table 1:** Clinical characteristics of family X members

Family member	Year of birth	Sex	Age at sarcoidosis diagnosis	Chest X ray stage^a^	Extra-thoracic sarcoidosis	Sarcoidosis duration (years)	Sarcoidosis treatment
IA	1925	M	41	4	no	30	not treated
IB	1932	F	43	1	skin	34	never treated
IIA	1955	M	35	4	PH	28	corticosteroid
IIB	1957	M	51	1	spinal cord	10	corticosteroid/cyclphosphamid

*M* male, *F* female; ^a^according to (17), *PH* pulmonary hypertension under specific vasodilatator treatment

*Members of family X unaffected by sarcoidosis (n = 2)* (Fig. 1). Two members of the family (IIC and IID), who were brother or sister of two patients, son or daughter of one patient and nephew or niece of the fourth patient, with no history of sarcoidosis were investigated in 2016. For both, clinical examination, chest X ray and blood biology including serum angiotensin converting enzyme were normal.

*Control volunteers (n = 4)*: Four normal volunteers, aged 69, 65, 48 and 53 with no history of sarcoidosis nor any known current disease served as normal controls.

### Genetic testing

Blood DNAs were obtained from three patients and tested for *NOD2 exon 8, IL17RA exon 11, KALRN exon 1 and EPHA2 exon 17* sequence variants. PCR reaction was performed as described in the Additional file 1.

### Targeted exome sequencing

Genomic DNA was captured using Agilent in-solution enrichment methodology (SureSelect Human Clinical Research Exome, Agilent) with the supplied biotinylated oligonucleotides probes library (Human Clinical Research Exome, Agilent), followed by paired-end 75 bases massively parallel sequencing on Illumina HiSEQ 4000. The DNA library was prepared as described in the Additional file 1.

### Screening of SNP variants identified by WES

SNP variants identified in family X upon WES screening were analyzed for a putative functional effect on the protein by using the SIFT and Polyphenv2 softwares (see in the Additional file 1). The minor allele frequencies (MAF) were evaluated by the ExAC online database (see in the Additional file 1, Bioinformatics and Statistical and functional evaluation of variants in silico). A MAF less than 0.01 suggest a rare variant and not a common polymorphism.

### Peripheral blood mononuclear cells isolation, culture and stimulations

Twenty mL of total blood sampled in EDTA tubes for routine hematology analysis were collected. Peripheral blood mononuclear cells (PBMCs) were isolated and differentiated in macrophages as described in the Additional file 1. Macrophages were then stimulated with either saline serum (Ctrl), or 10 μg/mL of N-Acetylmuramyl-L-alanyl-D-isoglutamine hydrate (MDP, muramyl dipeptide) (Sigma-Aldrich France), or 1 μg/ml Lipopolysaccharides from *Escherichia coli* 0111: B4 (LPS) (Sigma-Aldrich) for 1 h for the NF-κB activation study or 24 h for the transcriptional study.

### NF-κB activation

Activation of the different NF-κB family members (p50, p52, p65, c-Rel and RelB) was assayed using the TransAM® NF-κB Family kit (Active Motif, Belgium) according to the manufacturer’s protocol.

### RNA extraction and RT-QPCR

RNA were prepared as described in Additional file 1. Quantitative PCRs were performed in presence of ABsolute QPCR Mix (Thermo Fisher Scientific) with primer sets specific to *TNF-A, IL-8, IL-6, NOD2* and *IL17RA* (see in the Additional file 1: Table S1).

## Results

### Sarcoidosis presentation, outcome and treatment

Sarcoidosis presentation, outcome and treatment are shown in Table 1. Sarcoidosis was diagnosed in 35, 41, 43 and 51-year-old patients. Typical bilateral hilar lymphadenopathy was evidenced on thorax imaging in all four cases with lung infiltration in two cases. Bilateral upper lobes fibrosis was seen in two cases, associated with pulmonary hypertension in only one case. Other manifestations were respectively skin lesions for IB and spinal cord localization for IIB. In three cases (IB, IIA, IIB), there was a prolonged course (respectively 35, 27 and 9 years) with no healed case till now. Patient IB with a well-tolerated chronic sarcoidosis has upper lobes pulmonary fibrosis with calcification of hilar and mediastinal lymph nodes. She received no systemic therapy for sarcoidosis, except treatment for recurrent infectious exacerbations. The exact duration of the disease course is unknown. For IIA, a corticosteroid treatment was given after 20 years due to lung progression and occurrence of lung fibrosis. There was a good response with glucocorticoids which could be tapered until 7 mg/d with no evidence of residual activity on recent investigations. Eventually, for IIB, because of spinal cord involvement, a treatment associating high doses of corticosteroids and cyclophosphamide was given with a favourable response. Now, treatment consists in low doses of prednisone without relapse.

### NOD2 investigation

The *NOD2* variant (SNP - Ggc/Cgc – rs2066845 – position chr.16.50756540 – transcript NM_022162) identified in this family is a missense mutation previously described in the context of Crohn’s disease. It consists of a G908R substitution located in exon 8. The minor allele frequency as suggested by the ExAC database is 0,009917 (http://exac.broadinstitute.org/). In family X, this variant was found in the three patients with sarcoidosis and one among two offsprings free of the disease. No control had NOD2 mutation. In addition, selective extraction of rare variants from WES data allowed us to identify three other single nucleotide variants in the three patients with sarcoidosis. These SNPs target the coding regions of the *IL17RA*, *KALRN* and *EPHA2* genes, causing either missense (*IL17RA, EPHA2*) or nonsense (*KALRN*) variants. Data on the various SNPs are summarized in Table 2. In the series of 9 families screened by WES and including family X, a total of 7 deleterious and /or rare variants was observed in the three genes, either *EPHA2* (3 variants in 3 families), *KALRN* (2 variants in 2 families) and *IL17RA* (2 variants in 2 families) (Table 3). The rs139787163 variant of *EPHA2* (p.Ala951Thr) was observed two times despite a very low MAF value (ExAC: 0.000346).

**Table 2:** Description of heterozygous variants identified by whole-exome-sequencing in the family X

Gene variant	*NOD2 2722G > C* *exon 8* NM_022162	*IL17RA 958 T > C* *exon 11* NM_014339.6	*KALRN 28C > T* *exon 1* NM_007064.3	*EPHA2 2875G > A* *exon 17* NM_004431.3
Chr.	16	22	3	1
Position	50,756,540	17,586,757	124,303,696	16,451,766
QUAL	2521	3844	4578	10,401
Deph	278	370	528	618
rs ID number	rs2066845	rs140221307	rs56407180	rs139787163
Single nucleotide variant	missense	missense	nonsense	missense
PolyPhen2 prediction	0.986^a^	0.972^a^	STOP^a^	0.828^a^
SIFT	0.01	0^a^	STOP^a^	0.05^a^
EXaC global MAF	0.009917	0.001801	0.002382	0.00346
Protein	NOD2 G908R	IL17RA W320R	–	EPHA2 A959T
Prior associations with disease	Crohn’s disease, Psoriatic arthritis, Blau syndrome	Familial Candidiasis	–	Age-related cortical cataract
Patient IB	Het	Het	Het	Het
Patient IIA	Het	Het	Het	Het
Patient IIB	Het	Het	Het	Het
Patient IIC	Het	A	A	A
Patient IID	A	A	A	A

*Het* heterozygous, *A* minor allele (C) absent, *Chr* chromosome, *SNP* single nucleotide polymorphism; Depth represents the number of reads identifying the SNP variant. QUAL., a quality parameter measuring the probability p that the observation of the variant is due to chance (for ex: QUAL = n, *p* = 1/n). It includes the DEPTH parameter and the coverage of the genomic sequence. The in silico functional evaluation of SNP variants was performed by using bioinformatics softwares SIFT, PolyPhen-2. The value indicating a putative pathogenic effect are near of 0 for SIFT and near of 1 for Polyphenv2, as indicated by the ^a^. The minor allele frequency of SNP variants were evaluated by using the ExAC online database (http://exac.broadinstitute.org/). A global MAF including all ethnic origins lower than 0.01 suggest a rare variation and not a common polymorphism

**Table 3:** Variants identified in other SARCFAM families

GENE	rs	Exon	Codon	TYPE	EFFECT	SIFT	POLYPHEN	Accession	Number of family	MAF (ExAC)	NUCLEOTIDE	ChR	POSITION	DEPTH mini
EPHA2	rs139787163	3	959	missense	Ala959Thr	0,05	0,828	NM_004431.3	Family X	0.000346	Gcc/Acc	1	16,451,766	48
EPHA2	rs150790360	3	165	missense	Val165met	0	0,062	NM_004431	1	0.001535	Gtg/Atg	1	16,475,203	157
EPHA2	rs139787163	17	959	missense	Ala959Thr	0,05	0,828	NM_004431	1	0.000346	Gcc/Acc	1	16,451,766	48
KALRN	rs56407180	1	10	nonsense	Arg10ter	STOP	GAIN	NM_007064.3	Family X	0.002382	c.28C > T	3	124,303,696	528
KALRN	rs35298864	38	1930	missense	Arg1930Met	0	0,891	NM_001024660	1	0.007276	aGg/aTg	3	124,369,782	178
IL17RA	rs140221307	11	320	missense	Trp320Arg	0	0,972	NM_014339.6	Family X	0.001801	c.958 T > C	22	17,586,757	370
IL17RA	rs41323645	13	691	missense	Ala691Thr	0,35	0,926	NM_014339.6	1	0.3023	c.2071G > A	22	17,590,180	160
Additional informations on IL17R														
IL17RB	rs2232346	9	278	missense	Phe278Leu	0,65	0	NM_018725.3	Family X	0.01915	c.832 T > C	3	53,892,830	141
IL17RC	nd	6	nd	splice donor	frameshift	splicing	defect		1	nd	c.790 + 1G > T	3	9,962,287	155

### NFκB activation is reduced in sarcoidosis patients with NOD2 G208R mutation

**Fig. 2:**
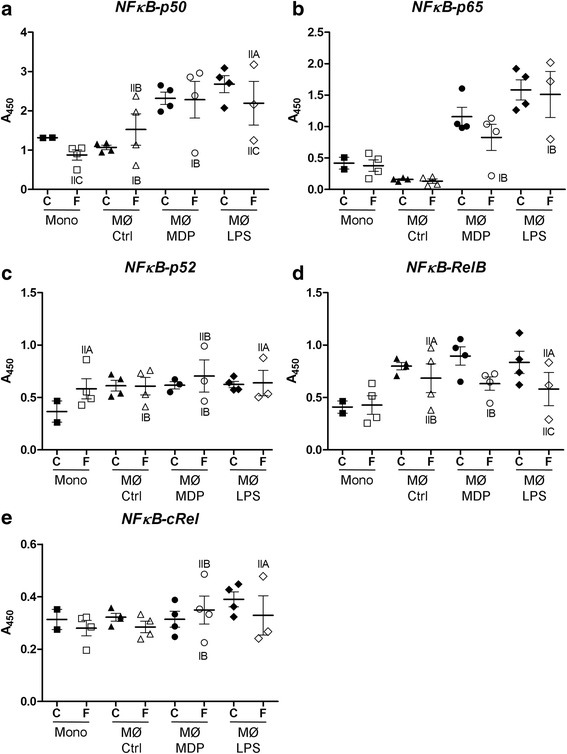
NF-κB activation is decreased in untreated NOD2 G208R sarcoidosis patient. NFκB activation (p50 (**a**), p65 (**b**), p52 (**c**), RelB (**d**), cRel (**e**)) (measured as OD450 nm) was assessed by TransAM NFκB kit (Active Motif) in control (C) and family X (F) monocytes (Mono) or monocytes derived macrophages (MФ) in basal condition (Ctrl) or treated with MDP or LPS for 1 h

Activation of NOD2 promotes its binding to RIP2 kinase, triggering the complex activation and initiating the activation of multiple complexes, including MAP kinases and IκB kinases [21]. This activation allowed the release of active NF-κB/Rel complexes that translocate to the nucleus where they induce target gene expression involved in innate and adaptive immunity, inflammation, stress responses, B-cell development, and lymphoid organogenesis. In the present study, activation of different NF-κB family members (p50, p52, p65, c-Rel and RelB) was assayed in monocytes and macrophages in basal condition or in response to either MDP (specific NOD2 activator) or LPS from control subjects or family members. NF-κB p50 and p65 activations were increased in response to MDP and LPS stimulation in both control subjects and family patients, except the patient IB whose macrophages poorly responded to both treatments (Fig. 2a, b). Similarly, NF-κB p52 activation was less in macrophages from patient IB (Fig. 2c). No major differences were observed in NF-κB cRel and RelB activation in macrophages from control subjects or family members in stimulated by MDP or LPS (Fig. 2d, e).

**Fig. 3:**
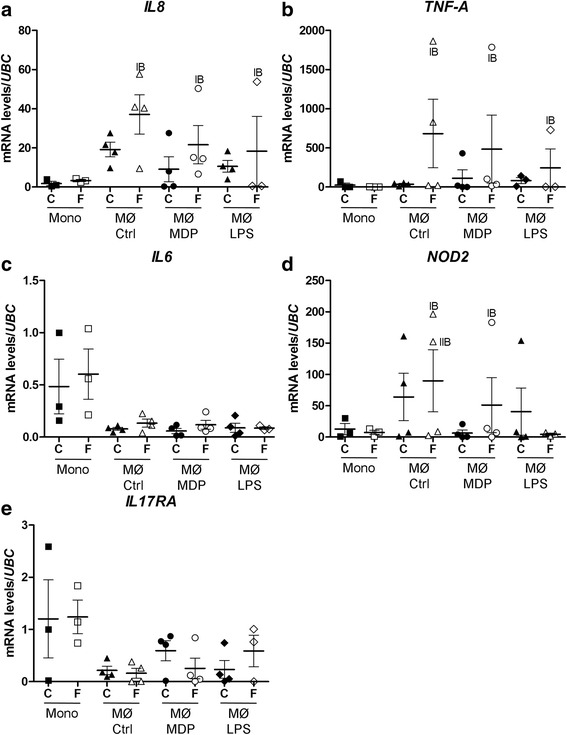
Up-regulation of *IL-8* and *TNF-A* expression in untreated NOD2 G208R sarcoidosis patient. *IL-8* (**a**), *TNF-A* (**b**), *IL-6*  (**c**),* NOD2* (**d**) and *IL17RA* (**e**) mRNA levels were assessed by RT-qPCR in control (C) and family X (F) monocytes (Mono) or monocytes derived macrophages (MФ) in basal condition (Ctrl) or treated with MDP or LPS for 24 h

To determine whether reduced NF-κB activation in untreated NOD2 G208R sarcoidosis patient (IB) decreases inflammatory cytokines expression, mRNA levels for *IL-8, TNF-A, IL-6* genes were assessed by quantitative RT-PCR (qRT-PCR) in monocytes and macrophages in basal condition or in response to either MDP or LPS from control subjects or family members. Strikingly, both *IL-8* and *TNF-A* mRNAs were expressed at high levels in macrophages from patient IB in basal and stimulated conditions compared to other family members and control subjects (Fig. 3a, b). By contrast, *IL-6* mRNA levels were similar in both groups in each culture conditions (Fig. 3c). In addition, NOD2 mRNA levels were increased in patient IB in basal condition and in response to MDP (Fig. 3d). By contrast, IL17RA were similar in both groups in each culture conditions (Fig. 3e).

## Discussion

To our knowledge, this is the first time that the *NOD2 2722G > C* (rs2066845) variant substituting a Glycine to Arginine at codon 908 in exon 8 in the leucine rich repeat (LRR) domain of the NOD2 protein is reported in a case of familial sarcoidosis with late and typical presentation of the disease. This variant was observed in an unaffected relative, suggesting that other genetic and/or epigenetic factors may contribute to the occurrence of the disease. Indeed, WES analysis discriminated affected versus unaffected individuals by three putative pathogenic SNP variants in the *IL17RA*, *KALRN* and *EPHA2* genes.

NOD2 is essential in regulating both inflammatory and immunologic homeostasis. *In silico* pathogenic evaluation (p) with the SIFT (*p* = 0.01) and POLYPHENv2 (PSIC score difference = 2.407) suggest a strong disturbing effect on the primary amino acid sequence raising the question of a significant impact in sarcoidosis pathogenesis. The role of the G908R mutation of the *NOD2* gene in sarcoidosis is controversial either with increase susceptibility for developing sarcoidosis [17, 18] or a minor role of the *2722G > C* variant in the pathogenesis of sarcoidosis [22–24]. In the present case of familial sarcoidosis, 3 out of 4 patients with the variant *2722G > C* developed sarcoidosis, while the other unaffected offspring did not carry the variant. Observation of a family member with NOD2 variant but no sarcoidosis is similar to the numerous individuals free of any disease, particularly of CD despite carrying the same variant, indicating that gene/environmental interactions and possibly gene/gene interactions (epistasis) are also essential [25]. Such association of sarcoidosis with the *NOD2 2722G > C* variant is certainly a very rare finding. It was not observed in Bello’s paper focused on adult patients with sarcoidosis involving eyes, skin and joints as in EOS and BS [16]. In our nine other families with at least 3 members affected, none was associated to any *NOD2* variant. Among our family collection, families with 3 or more affected members (*n* = 10) represent probably a small subset among sarcoidosis families with probably specific genetic predisposal or shared environmental factors.

It is of particular interest to observe the *NOD2 2722G > C* variant associated with a very typical presentation of sarcoidosis, all patients showing bilateral hilar lymphadenopathy and lymphatic distribution of lung micronodules distinct from EOS when lung involvement was present [26]. By contrast evidence of lung infiltration is very rare in EOS with a very different radiologic pattern and no lymphadenopathy [11]. Another question is the impact of NOD2 variant on the outcome which was characterized by a very long course in all patients, a condition usual in CD and perhaps associated to persistent abnormalities of microbiota induced by the variant. Indeed, deletion of *Nod2* in mice reduced bacterial activity in the gut, promoting an increased susceptibility to colonization by both the commensal microbiota and pathogenic bacteria, thus triggering intestinal inflammation [27]. In addition, *Nod2* deletion impairs autophagy in macrophages allowing intracellular survival of bacteria delaying bacterial clearance [28]. In our family, any confusion with CD either as main diagnosis or as comorbidity was ruled out by the absence of suggestive history of digestive tract disease particularly in one patient who had two colonoscopic examinations.

Both gain of function NOD2 mutant alleles like in BS and EOS and loss of function NOD2 mutant alleles like in most common CD and probably in our family with sarcoidosis might induce autoinflammatory disorders [29, 30]. During sarcoidosis, NF-κB is activated in site of organ involved [31–33]. In BS and EOS, gain of function NOD2 variants are associated with NF-κB activation. By contrast, we observed NFκB downregulation in our untreated patient as in CD patients with the *NOD2 2722G > C* variant. These data are supported by *in vitro* experiments showing that NFκB activity is reduced at baseline and in response to MDP in HEK293T cells expressing the *NOD2 2722G > C* variant [34–36]. Interestingly, NF-κB activity was upregulated in response to MDP in family X treated patients as well as the unaffected carrier of the variant, similarly to normal controls suggesting that treatments efficient against sarcoidosis activity may restore NF-κB activity altered by the presence of the *NOD2 2722G > C* variant. Indeed, patients treated by glucocorticoids responded well to the treatment, indicating that they were not refractory to corticoids [37] by comparison to Crohn disease patients [38]. Although increased NF-κB activity in patients with corticoids is obviously in conflict with what could be expected with anti-inflammatory drugs, previous studies showed that glucocorticoids could potentially contribute to pro-inflammatory activation by inducing the expression of the Macrophage migration inhibitory factor [39]. By contrast, in response to LPS stimulation, NF-κB activity was maintained in the untreated patient IB to similar levels observed in family X treated patients and normal controls, indicating that stimulation of NF-κB through NOD2- independent pathways remains possible. NF-κB is an important transcription factor for genes encoding cytokines like *IL-8* and *TNF-A*, this last one being a corner stone in sarcoidosis pathogenesis. In patient IB, despite a decrease in NF-κB activity, *IL-8* and *TNF-A* mRNA levels were increased at baseline and in stimulated conditions, indicating a chronic pro-inflammatory status in macrophages. As in Crohn disease patients with the *NOD2 2722G > C* variant, patient IB led to an apparent paradox: reduced NF-κB transactivating activity associated with more inflammation. Recently Strober et al... showed that in mice, activation of NF-κB in response to ligands to toll-like receptor 2 (TLR-2) is down-regulated by NOD2 [40]. In the absence of an efficient form of NOD2 the TLR signaling pathway is maintained, leading to increased production of inflammatory cytokines and the development of pathogenesis. Other data suggest that virulence and pathogens factors may trigger the NOD1/NOD2 signaling pathway indirectly by activating Rac1 [41]. Rac1 has been shown to upregulate NF-κB activity in a PAK1 (p21-activated kinase) dependent manner by stimulating the nuclear translocation of p65 subunit of NF-κB [42]. In addition, Hedl et al showed that acute stimulation of NOD2 triggered in human macrophages the secretion of anti-inflammatory cytokines, including IL-10 and IL1ra [43]. Thus, we could hypothesize that in *NOD2 2722G > C* patients, an imbalance occurs in favour of a pro-inflammatory phenotype.

One of the major issue was to find any genetic data which might discriminate family X members expressing clinically the disease from the others. Remarkably, in addition to *NOD2 2722G > C*, three single nucleotide variants for the *IL17RA*, *KALRN* and *EPHA2* genes were found in the family X patients with sarcoidosis. By comparison, as shown in Table 3, screening of the nine families by WES analysis showed the presence of single deleterious variants in a total of 7 families out of 9, one of them (*EPHA2* – rs139787163) being common in two distinct families despite a very low minor allele frequency (MAF = 0.000346).

The *IL17RA 958 T > C* variant modifies the amino acid sequence in the extracellular domain of the IL17RA, possibly altering its function. IL17RA (interleukin 17A receptor) is capable to form a heterodimer complex to bind both interleukin 17A and IL-25. IL17RA plays a pathogenic role in many inflammatory and autoimmune diseases, including rheumatoid arthritis, psoriasis, *Candida albicans* infection and Crohn disease [44–46]. Activation of IL17RA leads to induction of expression of inflammatory cytokines such as CXCL1, CXCL8/IL8 and IL6 through the trans-activation of NF-κB, AP-1 and C/EBPβ. Involvement of IL17 signalling was shown in sarcoidosis [47]. Notably, IL-17–producing T cells are increased in peripheral blood and lungs of subjects with sarcoidosis compared with controls [48]. In addition, IL-17A plays a major role in granuloma formation in response to mycobacterial infections in mice [49, 50]. Likewise Th17 cells have been implicated in the development of Crohn’s disease [51]. Notably, McGovern et al... showed that IL17RA genetic variants increased susceptibility to inflammatory bowel disease pathogenesis and demonstrated the cumulative risk of IL17RA variants with genes of the IL23/IL17 pathway in the development of CD [46]. Interestingly, Kurdy et al demonstrated that IL17 is directly regulated at the transcriptional level by a complex including Rac1, Tiam1 and RORχt (RAR-related orphan receptor gamma), the master transcription and differentiation factor of Th17 cells [52].

The *EPHA2 2875G > A* variant is located within the conserved cytoplasmic domain of the receptor. This variant was associated with age-related cortical cataract [53–56]. Erythropoietin-producing hepatoma (Eph) receptors are a family of receptor tyrosine kinases that can bind ephrin ligands. EphA receptors are expressed on CD4^+^ and CD8^+^ T cells, dendritic cells (DCs), and Langherans cells [57, 58] and are involved in T cell function [59]. Eph receptors are involved in axon guidance, vascularization, tissue assembly, and cell adhesion and migration [60–63]. Khounlothm et al... showed that EphA receptors play a role in the pathogenesis of *M. tuberculosis* infection by reducing the migration of T cells and DCs to the site of infection, producing an environment that favours bacterial persistence [64]. Activated EPHA2 interacts with Rho family GEF in endosomes and play a role in ephrin-dependant Rac1 activation [65]. Interestingly, variants for EPH receptor family members have been associated with inflammatory bowel diseases. Hafner et al showed an increased expression of Eph-B2 that increased epithelial cell mobility in the intestinal epithelium of Morbus Crohn patients [66].

The KALRN 28C > T variant is a non-sense variant targeting Kalirin, a multidomain guanine nucleotide exchange factor (GEF) for small GTP-binding proteins of the Rho family. Multiple kalirin isoforms containing different combinations of functional domains are predominantly expressed in brain, except KALRN 9 that is more widely expressed [67]. Kalirin is known to be involved in active remodeling of synapses and dendritic maturation in early development [68]. Kalirin was also shown to play a neuroprotective role during inflammation of the central nervous system by inhibiting iNOS activity [69]. In smooth muscle cells, Kalirin interacts with Rac1, promoting SMC migration and proliferation [70]. Thus, it appears that both EPHA2 and KALRN are implicated in dendritic cell maturation and migration. Dendritic cells have been implicated in sarcoidosis [71]. Mathew et al. showed that myeloids DCs function was reduced in sarcoidosis patients, possibly contributing to susceptibility and persistence of the chronic inflammation [72]. Interestingly in family X, member IIC who carries the *NOD2 2722G > C* variant only*,* did not develop sarcoidosis. These data suggest that the *NOD2 2722G > C* variant was not sufficient to trigger pulmonary sarcoidosis by itself and it is the presence of other variants (*IL17RA 958 T > C, EPHA2 2875G > A* and *KALRN 28C > T)* that contribute to the pathology in patients with pulmonary sarcoidosis of family X, perhaps by enhancing the development and chronicity of pulmonary sarcoidosis in family X.

Taken together, our data suggest a functional and pathogenic link between EPHA2, KALRN and IL17RA in the occurrence of the disease of family X sarcoidosis patients, acting in addition to the NOD2 functional defect.

## Conclusions

For the first time in the literature, we described the presence of the *NOD2 2722G > C* variant in a case of familial sarcoidosis. Our finding further establishes that the *NOD2 2722G > C* variant in combination with variants for *IL17RA*, *EPHA2* and *KALRN* genes could play a significant role in the development of sarcoidosis. Future functional studies are required to reveal the causal regulatory variation of the various loci and the immunogenetic basis related to sarcoidosis.

## Additional file

Additional file 1: Contains supplementary data for the Material and Methods section. (DOC 64 kb)

## References

[1] Pacheco Y (2011). Sarcoidosis and genetics. Rev Mal Respir.

[2] Sverrild A, Backer V, Kyvik KO, Kaprio J, Milman N, Svendsen CB, Thomsen SF (2008). Heredity in sarcoidosis: a registry-based twin study. Thorax.

[3] Li Y, Pabst S, Lokhande S, Grohe C, Wollnik B (2009). Extended genetic analysis of BTNL2 in sarcoidosis. Tissue Antigens.

[4] Rybicki BA, Walewski JL, Maliarik MJ, Kian H, Iannuzzi MC (2005). The BTNL2 gene and sarcoidosis susceptibility in African Americans and Whites. Am J Hum Genet.

[5] Valentonyte R, Hampe J, Huse K, Rosenstiel P, Albrecht M, Stenzel A, Nagy M, Gaede KI, Franke A, Haesler R (2005). Sarcoidosis is associated with a truncating splice site mutation in BTNL2. Nat Genet.

[6] Hofmann S, Franke A, Fischer A, Jacobs G, Nothnagel M, Gaede KI, Schurmann M, Muller-Quernheim J, Krawczak M, Rosenstiel P, Schreiber S (2008). Genome-wide association study identifies ANXA11 as a new susceptibility locus for sarcoidosis. Nat Genet.

[7] Fischer A, Schmid B, Ellinghaus D, Nothnagel M, Gaede KI, Schurmann M, Lipinski S, Rosenstiel P, Zissel G, Hohne K (2012). A novel sarcoidosis risk locus for Europeans on chromosome 11q13.1. Am J Respir Crit Care Med.

[8] Muller-Quernheim J, Schurmann M, Hofmann S, Gaede KI, Fischer A, Prasse A, Zissel G, Schreiber S (2008). Genetics of sarcoidosis. Clin Chest Med.

[9] Fischer A, Ellinghaus D, Nutsua M, Hofmann S, Montgomery CG, Iannuzzi MC, Rybicki BA, Petrek M, Mrazek F, Pabst S (2015). Identification of Immune-Relevant Factors Conferring Sarcoidosis Genetic Risk. Am J Respir Crit Care Med.

[10] Ogura Y, Bonen DK, Inohara N, Nicolae DL, Chen FF, Ramos R, Britton H, Moran T, Karaliuskas R, Duerr RH (2001). A frameshift mutation in NOD2 associated with susceptibility to Crohn's disease. Nature.

[11] Caso F, Galozzi P, Costa L, Sfriso P, Cantarini L, Punzi L (2015). Autoinflammatory granulomatous diseases: from Blau syndrome and early-onset sarcoidosis to NOD2-mediated disease and Crohn's disease. RMD Open.

[12] Rose CD, Pans S, Casteels I, Anton J, Bader-Meunier B, Brissaud P, Cimaz R, Espada G, Fernandez-Martin J, Hachulla E (2015). Blau syndrome: cross-sectional data from a multicentre study of clinical, radiological and functional outcomes. Rheumatology (Oxford).

[13] Sidiq T, Yoshihama S, Downs I, Kobayashi KS (2016). Nod2: A Critical Regulator of Ileal Microbiota and Crohn's Disease. Front Immunol.

[14] Rybicki BA, Hirst K, Iyengar SK, Barnard JG, Judson MA, Rose CS, Donohue JF, Kavuru MS, Rabin DL, Rossman MD (2005). A sarcoidosis genetic linkage consortium: the sarcoidosis genetic analysis (SAGA) study. Sarcoidosis Vasc Diffuse Lung Dis.

[15] Design of a case control etiologic study of sarcoidosis (ACCESS) (1999). ACCESS Research Group. J Clin Epidemiol.

[16] Bello GA, Adrianto I, Dumancas GG, Levin AM, Iannuzzi MC, Rybicki BA, Montgomery C (2015). Role of NOD2 Pathway Genes in Sarcoidosis Cases with Clinical Characteristics of Blau Syndrome. Am J Respir Crit Care Med.

[17] Gazouli M, Koundourakis A, Ikonomopoulos J, Gialafos EJ, Rapti A, Gorgoulis VG, Kittas C (2006). CARD15/NOD2, CD14, and toll-like receptor 4 gene polymorphisms in Greek patients with sarcoidosis. Sarcoidosis Vasc Diffuse Lung Dis.

[18] Schurmann M, Valentonyte R, Hampe J, Muller-Quernheim J, Schwinger E, Schreiber S (2003). CARD15 gene mutations in sarcoidosis. Eur Respir J.

[19] Pacheco Y, Calender A, Israel-Biet D, Roy P, Lebecque S, Cottin V, Bouvry D, Nunes H, Seve P, Perard L (2016). Familial vs. sporadic sarcoidosis: BTNL2 polymorphisms, clinical presentations, and outcomes in a French cohort. Orphanet J Rare Dis.

[20] Statement on sarcoidosis. Joint Statement of the American Thoracic Society (ATS), the European Respiratory Society (ERS) and the World Association of Sarcoidosis and Other Granulomatous Disorders (WASOG) adopted by the ATS Board of Directors and by the ERS Executive Committee, February 1999. Am J Respir Crit Care Med. 1999(160):736–55. 10.1164/ajrccm.160.2.ats4-99 10430755

[21] Corridoni D, Arseneau KO, Cifone MG, Cominelli F (2014). The dual role of nod-like receptors in mucosal innate immunity and chronic intestinal inflammation. Front Immunol.

[22] Sato H, Williams HR, Spagnolo P, Abdallah A, Ahmad T, Orchard TR, Copley SJ, Desai SR, Wells AU, du Bois RM, Welsh KI (2010). CARD15/NOD2 polymorphisms are associated with severe pulmonary sarcoidosis. Eur Respir J.

[23] Ho LP, Merlin F, Gaber K, Davies RJ, McMichael AJ, Hugot JP (2005). CARD 15 gene mutations in sarcoidosis. Thorax.

[24] Milman N, Nielsen OH, Hviid TV, Fenger K (2007). CARD15 single nucleotide polymorphisms 8, 12 and 13 are not increased in ethnic Danes with sarcoidosis. Respiration.

[25] Caruso R, Warner N, Inohara N, Nunez G (2014). NOD1 and NOD2: signaling, host defense, and inflammatory disease. Immunity.

[26] Nunes H, Uzunhan Y, Gille T, Lamberto C, Valeyre D, Brillet PY (2012). Imaging of sarcoidosis of the airways and lung parenchyma and correlation with lung function. Eur Respir J.

[27] Petnicki-Ocwieja T, Hrncir T, Liu YJ, Biswas A, Hudcovic T, Tlaskalova-Hogenova H, Kobayashi KS (2009). Nod2 is required for the regulation of commensal microbiota in the intestine. Proc Natl Acad Sci U S A.

[28] Lapaquette P, Bringer MA, Darfeuille-Michaud A (2012). Defects in autophagy favour adherent-invasive Escherichia coli persistence within macrophages leading to increased pro-inflammatory response. Cell Microbiol.

[29] Tigno-Aranjuez JT, Asara JM, Abbott DW (2010). Inhibition of RIP2’s tyrosine kinase activity limits NOD2-driven cytokine responses. Genes Dev.

[30] Zurek B, Proell M, Wagner RN, Schwarzenbacher R, Kufer TA (2012). Mutational analysis of human NOD1 and NOD2 NACHT domains reveals different modes of activation. Innate Immun.

[31] Conron M, Bondeson J, Pantelidis P, Beynon HL, Feldmann M, duBois RM, Foxwell BM (2001). Alveolar macrophages and T cells from sarcoid, but not normal lung, are permissive to adenovirus infection and allow analysis of NF-kappa b-dependent signaling pathways. Am J Respir Cell Mol Biol.

[32] Drent M, van den Berg R, Haenen GR, van den Berg H, Wouters EF, Bast A (2001). NF-kappaB activation in sarcoidosis. Sarcoidosis Vasc Diffuse Lung Dis.

[33] Semenzato G, Bortoli M, Brunetta E, Agostini C (2005). Immunology and pathophysiology. Sarcoidosis.

[34] Bonen DK, Ogura Y, Nicolae DL, Inohara N, Saab L, Tanabe T, Chen FF, Foster SJ, Duerr RH, Brant SR (2003). Crohn's disease-associated NOD2 variants share a signaling defect in response to lipopolysaccharide and peptidoglycan. Gastroenterology.

[35] Inohara N, Ogura Y, Fontalba A, Gutierrez O, Pons F, Crespo J, Fukase K, Inamura S, Kusumoto S, Hashimoto M (2003). Host recognition of bacterial muramyl dipeptide mediated through NOD2. Implications for Crohn's disease. J Biol Chem.

[36] Chamaillard M, Philpott D, Girardin SE, Zouali H, Lesage S, Chareyre F, Bui TH, Giovannini M, Zaehringer U, Penard-Lacronique V (2003). Gene-environment interaction modulated by allelic heterogeneity in inflammatory diseases. Proc Natl Acad Sci U S A.

[37] Schuliga M (2015). NF-kappaB Signaling in Chronic Inflammatory Airway Disease. Biomol Ther.

[38] Niess JH, Klaus J, Stephani J, Pfluger C, Degenkolb N, Spaniol U, Mayer B, Lahr G, von Boyen GB (2012). NOD2 polymorphism predicts response to treatment in Crohn's disease--first steps to a personalized therapy. Dig Dis Sci.

[39] Calandra T, Bernhagen J, Metz CN, Spiegel LA, Bacher M, Donnelly T, Cerami A, Bucala R (1995). MIF as a glucocorticoid-induced modulator of cytokine production. Nature.

[40] Watanabe T, Kitani A, Murray PJ, Strober W (2004). NOD2 is a negative regulator of Toll-like receptor 2-mediated T helper type 1 responses. Nat Immunol.

[41] Keestra AM, Baumler AJ (2014). Detection of enteric pathogens by the nodosome. Trends Immunol.

[42] Frost JA, Swantek JL, Stippec S, Yin MJ, Gaynor R, Cobb MH (2000). Stimulation of NFkappa B activity by multiple signaling pathways requires PAK1. J Biol Chem.

[43] Hedl M, Abraham C (2011). Secretory mediators regulate Nod2-induced tolerance in human macrophages. Gastroenterology.

[44] Chen K, Eddens T, Trevejo-Nunez G, Way EE, Elsegeiny W, Ricks DM, Garg AV, Erb CJ, Bo M, Wang T (2016). IL-17 Receptor Signaling in the Lung Epithelium Is Required for Mucosal Chemokine Gradients and Pulmonary Host Defense against K. pneumoniae. Cell Host Microbe.

[45] Conti HR, Bruno VM, Childs EE, Daugherty S, Hunter JP, Mengesha BG, Saevig DL, Hendricks MR, Coleman BM, Brane L (2016). IL-17 Receptor Signaling in Oral Epithelial Cells Is Critical for Protection against Oropharyngeal Candidiasis. Cell Host Microbe.

[46] McGovern DP, Rotter JI, Mei L, Haritunians T, Landers C, Derkowski C, Dutridge D, Dubinsky M, Ippoliti A, Vasiliauskas E (2009). Genetic epistasis of IL23/IL17 pathway genes in Crohn's disease. Inflamm Bowel Dis.

[47] Sakthivel P, Bruder D (2017). Mechanism of granuloma formation in sarcoidosis. Curr Opin Hematol.

[48] Ten Berge B, Paats MS, Bergen IM, van den Blink B, Hoogsteden HC, Lambrecht BN, Hendriks RW, Kleinjan A (2012). Increased IL-17A expression in granulomas and in circulating memory T cells in sarcoidosis. Rheumatology (Oxford).

[49] Okamoto Y, Umemura M, Yahagi A, O'Brien RL, Ikuta K, Kishihara K, Hara H, Nakae S, Iwakura Y, Matsuzaki G (2010). Essential role of IL-17A in the formation of a mycobacterial infection-induced granuloma in the lung. J Immunol.

[50] Lombard R, Doz E, Carreras F, Epardaud M, Le Vern Y, Buzoni-Gatel D, Winter N (2016). IL-17RA in Non-Hematopoietic Cells Controls CXCL-1 and 5 Critical to Recruit Neutrophils to the Lung of Mycobacteria-Infected Mice during the Adaptative Immune Response. PLoS One.

[51] Weaver CT, Elson CO, Fouser LA, Kolls JK (2013). The Th17 pathway and inflammatory diseases of the intestines, lungs, and skin. Annu Rev Pathol.

[52] Kurdi AT, Bassil R, Olah M, Wu C, Xiao S, Taga M, Frangieh M, Buttrick T, Orent W, Bradshaw EM (2016). Tiam1/Rac1 complex controls Il17a transcription and autoimmunity. Nat Commun.

[53] Celojevic D, Abramsson A, Seibt Palmer M, Tasa G, Juronen E, Zetterberg H, Zetterberg M (2016). EPHA2 Polymorphisms in Estonian Patients with Age-Related Cataract. Ophthalmic Genet.

[54] Tan W, Hou S, Jiang Z, Hu Z, Yang P, Ye J (2011). Association of EPHA2 polymorphisms and age-related cortical cataract in a Han Chinese population. Mol Vis.

[55] Hattersley K, Laurie KJ, Liebelt JE, Gecz J, Durkin SR, Craig JE, Burdon KP (2010). A novel syndrome of paediatric cataract, dysmorphism, ectodermal features, and developmental delay in Australian Aboriginal family maps to 1p35.3-p36.32. BMC Med Genet.

[56] Shiels A, Bennett TM, Knopf HL, Maraini G, Li A, Jiao X, Hejtmancik JF (2008). The EPHA2 gene is associated with cataracts linked to chromosome 1p. Mol Vis.

[57] Aasheim HC, Delabie J, Finne EF (2005). Ephrin-A1 binding to CD4+ T lymphocytes stimulates migration and induces tyrosine phosphorylation of PYK2. Blood.

[58] de Saint-Vis B, Bouchet C, Gautier G, Valladeau J, Caux C, Garrone P: Human dendritic cells express neuronal Eph receptor tyrosine kinases: role of EphA2 in regulating adhesion to fibronectin. Blood 2003, 102:4431–4440. 10.1182/blood-2003-02-0500 12907451

[59] Wohlfahrt JG, Karagiannidis C, Kunzmann S, Epstein MM, Kempf W, Blaser K, Schmidt-Weber CB (2004). Ephrin-A1 suppresses Th2 cell activation and provides a regulatory link to lung epithelial cells. J Immunol.

[60] Nakamoto M (2000). Eph receptors and ephrins. Int J Biochem Cell Biol.

[61] Brantley-Sieders DM, Caughron J, Hicks D, Pozzi A, Ruiz JC, Chen J (2004). EphA2 receptor tyrosine kinase regulates endothelial cell migration and vascular assembly through phosphoinositide 3-kinase-mediated Rac1 GTPase activation. J Cell Sci.

[62] Cheng N, Brantley DM, Liu H, Lin Q, Enriquez M, Gale N, Yancopoulos G, Cerretti DP, Daniel TO, Chen J (2002). Blockade of EphA receptor tyrosine kinase activation inhibits vascular endothelial cell growth factor-induced angiogenesis. Mol Cancer Res.

[63] Coulthard MG, Lickliter JD, Subanesan N, Chen K, Webb GC, Lowry AJ, Koblar S, Bottema CD, Boyd AW (2001). Characterization of the Epha1 receptor tyrosine kinase: expression in epithelial tissues. Growth Factors.

[64] Khounlotham M, Subbian S, Smith R, Cirillo SL, Cirillo JD (2009). Mycobacterium tuberculosis interferes with the response to infection by inducing the host EphA2 receptor. J Infect Dis.

[65] Boissier P, Chen J, Huynh-Do U (2013). EphA2 signaling following endocytosis: role of Tiam1. Traffic.

[66] Hafner C, Meyer S, Langmann T, Schmitz G, Bataille F, Hagen I, Becker B, Roesch A, Rogler G, Landthaler M, Vogt T (2005). Ephrin-B2 is differentially expressed in the intestinal epithelium in Crohn's disease and contributes to accelerated epithelial wound healing in vitro. World J Gastroenterol.

[67] McPherson CE, Eipper BA, Mains RE (2002). Genomic organization and differential expression of Kalirin isoforms. Gene.

[68] Yan Y, Eipper BA, Mains RE (2015). Kalirin-9 and Kalirin-12 Play Essential Roles in Dendritic Outgrowth and Branching. Cereb Cortex.

[69] Ratovitski EA, Alam MR, Quick RA, McMillan A, Bao C, Kozlovsky C, Hand TA, Johnson RC, Mains RE, Eipper BA, Lowenstein CJ (1999). Kalirin inhibition of inducible nitric-oxide synthase. J Biol Chem.

[70] Wu JH, Fanaroff AC, Sharma KC, Smith LS, Brian L, Eipper BA, Mains RE, Freedman NJ, Zhang L (2013). Kalirin promotes neointimal hyperplasia by activating Rac in smooth muscle cells. Arterioscler Thromb Vasc Biol.

[71] Agah R, Malloy B, Kerner M, Mazumder A (1989). Generation and characterization of IL-2-activated bone marrow cells as a potent graft vs tumor effector in transplantation. J Immunol.

[72] Mathew S, Bauer KL, Fischoeder A, Bhardwaj N, Oliver SJ (2008). The anergic state in sarcoidosis is associated with diminished dendritic cell function. J Immunol.

