# Supplementary material for: G908R NOD2 variant in a family with sarcoidosis
Source: Respir Res. 2018 Mar 20;19:44. doi: 10.1186/s12931-018-0748-5 (PMC5859391; doi:10.1186/s12931-018-0748-5)
Supplement: Supplementary file 1 — Contains supplementary data for the Material and Methods section. (DOC 64 kb) [file 12931_2018_748_MOESM1_ESM.doc]

G908R NOD2 variant in a family with sarcoidosis

Valérie Besnard^1^*, Alain Calender^2^*, Diane Bouvry^3^*, Yves Pacheco^4,5^, Catherine Chapelon-Abric, Florence Jeny ^1,3^, Hilario Nunes^1,3^, Carole Planès, Dominique Valeyre^1,3^

**Additionnal file 1**

**Additional file 1 contains supplementary data for the material and methods section**

**Material & Methods**

**Genetic testing**

PCR reaction was done using primers designed with the Primer3 software, and respectively:  *NOD2*_Exon8F (AAGTCTGTAATGTAAAGCCAC) and Exon8R (CCCAGCTCCTCCCTCTTC), *IL17RA*_Exon11F (GACATTCCCCAGTGGCCA) and Exon11R (CACATGCCAGGTTCTGTGC), *KALRN*_Exon1F (TGGCTCCCAGTAAGTCAGA) and Exon1R (GCGTTCTGTCTGGGAAACTG), and lastly *EPHA2*_Exon17F (TCTCCCAGGCCTACCTAACC) and Exon17R (GGGCTTGAGGTACCCTGTTT). The quality control of PCR products was performed on a LabChipGX system (PERKIN ELMER™) and the products purified by NucleoFast® 96 PCR Clean-up Kit from MACHEREY-NAGEL™. The sequencing was performed by Big Dye terminator v1.1 after purification with BigDye® XTerminator™ Purification Kit. Sequence delineation and base calling used an automated fluorescent DNA sequencer (Applied Biosystems™, model 3130xl).

**POLYPHEN and Sorting Intolerant From Tolerant (SIFT) data**

PolyPhen2 and SIFT were used to predict potential functional effects of identified SNPs in the Family X. PolyPhen2 searches for three-dimensional protein structures, multiple alignments of homologous sequences and amino acid contact information in several protein structure databases. Position-specific independent count (PSIC) scores were calculated for each of two variants, and the difference of PSIC scores of the two variants was measured. The higher a PSIC score difference, the higher the functional impact a particular amino acid substitution is likely to have. A PSIC score difference of 1.5 or above is considered to be damaging. SIFT, a sequence homology-based tool sorted intolerant from tolerant amino acid substitutions and predicted whether an amino acid substitution would have a phenotypic effect.

**Targeted exome sequencing**

Genomic DNA was captured using Agilent in-solution enrichment methodology (SureSelect Human Clinical Research Exome, Agilent) with the supplied biotinylated oligonucleotidesprobe library (Human Clinical Research Exome, Agilent), followed by paired-end 75 bases massively parallel sequencing on Illumina HiSEQ 4000. Sequence capture, enrichment and elution were performed according to manufacturer’s instruction and protocols (SureSelect, Agilent) without modification except that library preparation was performed with NEBNext® Ultra kit (New England Biolabs®). For library preparation 600 ng of each genomic DNA was fragmented by sonication and purified to yield fragments of 150-200 bp. Paired-end adaptor oligonucleotides from the NEB kit were ligated on repaired, A-tailed fragments then purified and enriched by 8 PCR cycles. 1200ng of these purified libraries were then hybridized to the SureSelect oligo probe capture library for 72 hr. After hybridization, washing, and elution, the eluted fraction was PCR-amplified with 9 cycles, purified and quantified by QPCR to obtain sufficient DNA template for downstream applications. Each eluted-enriched DNA sample was then sequenced on an Illumina HiSEQ 4000 as paired-end 75b reads. Image analysis and base calling is performed using Illumina Real Time Analysis (RTA 2.1.3) with default parameters. Library preparation, exome capture, sequencing and data analysis have been done by IntegraGen SA (Evry, France).

**Bioinformatics**

Two independent bioinformatics analyses were conducted, for the purpose of reducing the risk of missing a variant of interest. Both used the hg19 assembly version of the human genome. First, the Integragen© pipeline was used. Reads were mapped using Elandv2e, and duplicates were removed. The variant call was performed using CASAVA1.8. Regions with low mappability (QVCutoff < 90) and variants with a weak quality (10 for SNVs, 20 for indels), were filtered out. Variants were annotated with population databases (1000G, ESP, ExAC, plus an Integragen© inhouse database of exomes) and with score predictions (SIFT, Polyphen). Functional consequences of variants were predicted by Variant Effect Predictor. The familial analysis was done on each trio using Eris software (Integragen©). Second, our in-house pipeline (PASS) was used that follows the recommendations of the Broad Institute (https://www.broadinstitute.org/gatk/guide/best-practices). Reads were trimmed with Trimmomatic, and aligned using BWA-MEM. Duplicates were marked using PicardTools, and the alignments were realigned and recalibrated using GATK. The variant call and genotyping were performed using GATK HapoltypeCaller (gVCF mode) and Genotype GVCFs. Then, variants were recalibrated using GATK VariantRecalibrator. Variant annotation was done by snpEff/SnpSift, using both population (1000 Genomes, ExAC) and pathogenicity score (SIFT, Polyphenv2, Mutation Taster) databases. The familial analysis was performed with genoFilter (an in-house script). For this purpose, GATK Calculate Genotype Posteriors was first used with a pedigree file of the trio in order to reassign genotypes and genotype qualities. Only variants with balanced allele frequencies (maximum of imbalance of 0.35/0.65), and a genotype quality superior to 20 were considered.

**Statistical and functional evaluation of variants *in silico***

Gene sequences of the gene including known variations were downloaded from http://www.ensembl.org/ Homo_sapiens/Transcript/Variation_Transcript/. Predicted consequences of genetic variations were evaluated by using online bioinformatics tools such as SIFT, PolyPhen- 2 , and EX_Skip . The same information is compiled in ALAMUT© Visual software (from Interactive Biosoftware©) which allows us to know if the variants observed have already been described in recessive or dominant genetic diseases. A statistical analysis has been performed on these scores. VCF files were annotated with the dbNSFP v3.5 database . CADD (Combined Annotation Dependent Depletion) , SIFT and POLYPHEN ranked scores were recovered for the selected de novo and recessive variants and for all the variants in the same genes and in the same patients, a mean value of the scores was considered when variants affect several transcripts. The different scores being non-normally distributed, their median was compared in the two groups (selected de novo and recessive variant vs all other variants in the same genes) by a non-parametric Wilcoxon/Mann-Whitney test.

# Peripheral blood mononuclear cells isolation, culture and stimulations

Twenty mL of total blood sampled in EDTA tubes for routine hematology analysis were collected. Peripheral blood mononuclear cells (PBMCs) were isolated from total blood using centrifugation over a separation gradient. Monocytes were isolated from human PBMCs using the Pan Monocyte Isolation Kit (Miltenyi, France), and then seeded in non-coated 12-well plates at 1x10^6^ cells/well in RPMI 1640 GlutaMAX containing 2 mM of glutamine, 25 mM of HEPES, and 10% FBS. Monocytes were differentiated in macrophages in presence of 5 ng/ml M-CSF (BioTechne, France) for a week.

**RNA extraction and RT-QPCR**

# RNA was extracted from monocytes/macrophages using Trizol (Thermo Fisher Scientific, France) according to the manufacturer's protocol. Reverse transcription was performed on 1 µg of total RNA using Maxima Reverse Transcriptase (Thermo Fisher Scientific). Quantitative PCRs were performed in presence of ABsolute QPCR Mix (Thermo Fisher Scientific) with primer sets specific to **TNF-A, IL-8, IL-6, NOD2 and IL17RA (see Table E1)**. A probe set for *UBC* was used as the normalization standard. The PCR and relative quantifications were performed in a real-time PCR system (**StepOnePlus Real*-*Time PCR System**, Applied BioSystems).

| **Gene** | **Forward primer (5’- 3’)** | **Reverse primer (5’- 3)** |
| --- | --- | --- |
| *UBC* | CACTTGGTCCTGCGCTTGA | TTTTTTGGGAATGCAACAACTT |
| *IL8* | AGGACAAGAGCCAGGAAGAA | ACTGCACCTTCACACAGAGC |
| *IL6* | AATGAGGAGACTTGCCTGGT | GCAGGAACTGGATCAGGACT |
| *TNF-A* | CCCATGTTGTAGCAAACCCT | TGAGGTACAGGCCCTCTGAT |
| *NOD2* | TCCTGGAAGTCTGGCTCCGA | CCCAAACTCACAGCCTGCTC |
| *IL17RA* | CTGTCGCCACCAAGTGCAGATC | GAGTAGATGATCCAGACCTTCCTG |

Table S1: QPCR primers list.

1. Ng PC, Henikoff S. Sift: Predicting amino acid changes that affect protein function. *Nucleic acids research* 2003;31:3812-3814.

2. Adzhubei I, Jordan DM, Sunyaev SR. Predicting functional effect of human missense mutations using polyphen-2. *Current protocols in human genetics* 2013;Chapter 7:7-20.

3. Raponi M, Kralovicova J, Copson E, Divina P, Eccles D, Johnson P, Baralle D, Vorechovsky I. Prediction of single-nucleotide substitutions that result in exon skipping: Identification of a splicing silencer in brca1 exon 6. *Human mutation* 2011;32:436-444.

4. Liu X, Wu C, Li C, Boerwinkle E. Dbnsfp v3.0: A one-stop database of functional predictions and annotations for human nonsynonymous and splice-site snvs. *Human mutation* 2016;37:235-241.

5. Kircher M, Witten DM, Jain P, O'Roak BJ, Cooper GM, Shendure J. A general framework for estimating the relative pathogenicity of human genetic variants. *Nature genetics* 2014;46:310-315.
